# A first-order flexible ELA model based on geomorphic constraints

**DOI:** 10.1016/j.mex.2020.101173

**Published:** 2020-12-05

**Authors:** Durban G. Keeler, Summer Rupper, Joerg M. Schaefer

**Affiliations:** a Department of Geography, University of Utah, Salt Lake City, UT, USA; b Lamont-Doherty Earth Observatory, Columbia University, Palisades, NY, USA

**Keywords:** Glacier modeling, Climate reconstruction, Equilibrium line altitude

## Abstract

Alpine glaciers, with their valuable combination of highly sensitive response to climate and near-global extent, are powerful tools for investigating previous and present climate changes. They also represent critical water resources for areas around the globe, with the potential for far-reaching effects in a warming world. Advancements to understand and model glacial changes and the variables influencing them are therefore paramount. Many glacier models fall into one of two endmembers: either highly complex transient models requiring careful tuning of multiple parameters to individual glaciers, or basic empirical correlations of glacier area and length with few considerations for local and regional variations in characteristics. Here we detail a physical steady-state model for alpine glaciers relating directly to glacier mass balance (via the equilibrium line altitude) while retaining the simplicity of other morphology methods, and simultaneously including error estimates. We provide custom MATLAB functions as a user-friendly and generally-applicable method to estimate glacier equilibrium line altitudes from only a limited number of glacier bed topography and glacier width measurements. As a test of the model’s efficacy, we compare the model results for present-day glaciers in the Swiss Alps with previously published estimates of equilibrium line altitudes and intermediate model outputs.

• The method estimates glacier equilibrium line altitudes from a limited set of bed topography measurements and constraints on glacier width.
• The method is based on continuity equations, reducing the need for empirical coefficients tuned with measured data.
• The method uses Monte Carlo sampling and bootstrapping to generate uncertainty bounds on the equilibrium line altitude estimates.

## Graphical abstract

**Figure:**
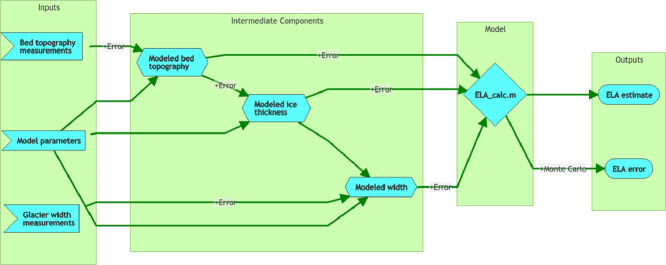

Specifications table

**Table:** 

Subject Area:	Earth and Planetary Sciences
More specific subject area:	Glaciology
Method name:	Equilibrium line altitude model based on geomorphic constraints
Name and reference of original method:	J. Oerlemans, Minimal glacier models, Utrecht Publishing & Archiving Service, Utrecht, 2008.
Resource availability:	Code and example data available at: https://github.com/durbank/ELA-model

## Introduction

Changes in glacier size and extent are fundamentally related to the mass balance of the glacier. An annual mass surplus (when net accumulation exceeds ablation) leads to glacier growth, while a deficit leads to glacier retreat. Such transitions can be visually drastic, with some glaciers changing by kilometers in response to minor perturbations in climate. A more comparable measure of the glacier response to climate change than glacier area or length changes is the concept of the equilibrium line altitude (ELA). The ELA is the boundary between the accumulation and ablation zones on a glacier and represents the elevation at which the annual amount of mass added through accumulation exactly equals the annual amount of mass lost through ablation [1]. As a direct measure of glacier mass balance, the ELA facilitates explicit comparisons of climate in space and time by accounting for dependencies on glacier size, extent, and shape, and by integrating the myriad variables that can drive changes in climate into a single comparable metric.

The most accurate method to determine the ELA is direct measurements of the distribution of accumulation and ablation on a glacier. Such measurements, however, are only available for a small subset of modern glaciers, and entirely absent for paleoglaciers. Several empirical proxy methods have therefore been developed to estimate ELA when direct measurements of mass balance are not possible. Some of the more widely used are the accumulation area ratio (AAR), the toe-to-headwall altitude ratio (THAR), the area-weighted mean altitude (AMA), and the median elevation of the glacier (MEG) [2], [3]. Such statistical methods are useful within many contexts. Braithwaite and Raper [3] for instance found an overall $R^{2}=0.99$ for 94 glaciers between steady-state ELA and one of the simplest ways to estimate ELA (MEG). Estimates of individual glaciers, however, can significantly diverge. Moreover, the empirical nature of these methods make them relatively easy to apply, but is also one of the primary inherent limitations on their applicability and interpretability. Because the empirical coefficients are derived from aggregate glacier data sets, these models are only valid for glaciers within the boundary conditions of the training data, typically with no a priori method to determine whether such an assumption is valid when applied to other regions or other periods of time. Such regionally-tuned coefficients can vary widely. AAR values among modern glaciers, for instance, vary between 0.22 and 0.72, resulting in a large range in possible ELA estimates depending on the value chosen [4]. The other empirical methods for ELA reconstructions suffer from similar concerns as the AAR method. Additionally, such methods provide no insight into the sensitivity or uncertainty of the estimates to glacier characteristics such as bed topography or areal distribution. All of these methods, although useful in many circumstances, highlight the need for additional progress to help better constrain ELA estimates in a robust, self-consistent manner and place the estimate within the context of model error, while still requiring minimum inputs.

**Fig. 1:**
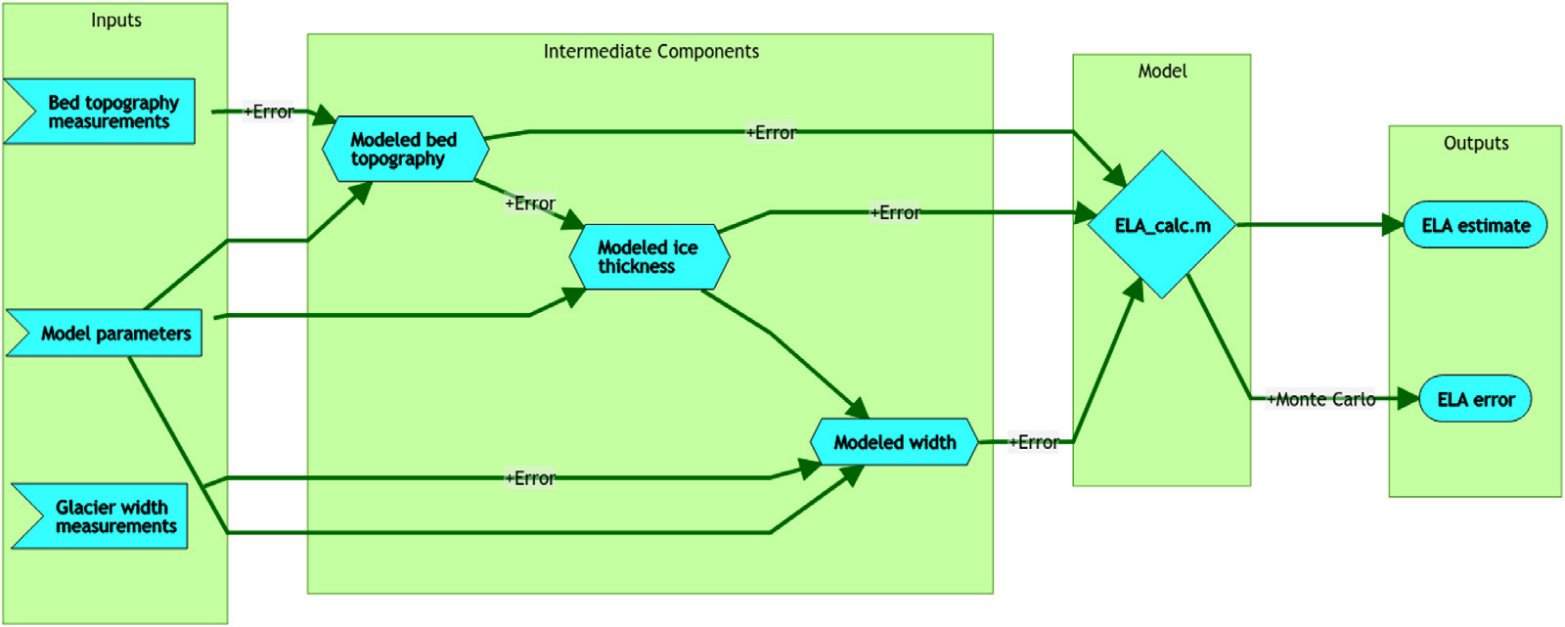
Graphical abstract describing the ELA modeling process used in this study.

This manuscript presents a method to reconstruct ELA estimates based on continuity equations, while only requiring estimates of bed topography, glacier length, and glacier width. The ELA model also generates intermediate results of continuous modeled bed topography, ice surface elevation, and glacier width along the length of the glacier (Fig. 1). An added strength of this model is that such intermediate outputs allow for increased diagnostics on model performance or troubleshooting. The required inputs for the model are similar to those required for simple geomorphic methods, but based on continuity equations rather than relying purely on empirical correlations while also accounting for physical errors in measurements. This method can equally apply to existing glaciers or paleo-glacier extents where glacial moraines are adequately preserved.

## Methodology

The presented model is largely derived from a simple linear glacier-length model presented in [5], with modifications specific to quantifying ELAs and ELA changes. The limited model inputs necessarily require simplifying assumptions that do not include all details pertinent to specific glaciers. Such details can be significant for some applications (e.g. dynamic modeling of glacier response, higher order surface energy and mass balance modeling, etc.), and other methods would be better suited to these circumstances. The proposed model is specifically intended to estimate the ELA of snow-fed, clean ice, temperate valley glaciers with relatively simple bed and areal geometries.

The ELA model also provides analytical constraints on the error associated with model outputs. Such uncertainties are fundamental in determining the significance and reliability of results, but rigorous physical uncertainties of ELA estimates are rarely presented in paleo-glacier research, either because such uncertainties are difficult to assign for geomorphic methods like THAR and AAR or because higher order models are sufficiently complex to challenge error propagation. Uncertainty estimates in this study are calculated based on Monte Carlo simulations of bootstrapped residuals of the input parameters. These uncertainties give insight into the range of plausible ELA values based on both uncertainty of input parameters and the ability of the model assumptions to accurately represent those inputs.

Other computational methods exist to estimate the ELA of paleoglaciers. Benn and Hulton [6] presents an Excel^TM^ spreadsheet to calculate the ice surface profiles of a former mountain glacier or ice cap, given bed topography and a yield stress. Pellitero et al. [7] provides a Python-based ArcGIS toolbox to automatically calculate glacier ELAs with a choice of methods (Accumulation-Area Ratio, Area-Altitude-Balance Ratio, Area-Altitude, or median elevation). The tool requires a DEM of the reconstructed glacier surface as input. Pellitero et al. [8] builds on [7] by adding the ability to estimate and interpolate a paleoglacier ice surface given the 3D bed topography and a center flowline. These methods represent important steps forward by incorporating ice flow laws and automating much of the process in ELA calculations in an accessible manner.

Similar to [6], [8], the presented ELA model also estimates the glacier surface based on centerline ice flow, given bed topography. It also aims to automate many of the steps in calculating an ELA to provide an easy-to-use and widely applicable method of ELA estimation. The main advantages of the presented ELA model are (1) less reliance on empirical relations which require tuning of coefficients and (2) the inclusion of robust uncertainty bounds based on Monte Carlo sampling. The decreased reliance on empirical correlations permits more widespread applicability without a priori knowledge and tuning of model coefficients. Such coefficients are determined regionally using glacier mass balance measurements from many glaciers in the area, even though nearby glacier values can diverge notably [2]. The proposed ELA model seeks to avoid these empirical complications by estimating the ELA directly from continuity equations. Another important advantage of the proposed ELA model is the incorporation of Monte Carlo bootstrapping. This permits estimation of robust uncertainties both from error in input parameters and from model limitations. These uncertainties give proper context to how accurate results are for specific glaciers, as well as how sensitive these glaciers are to various inputs. Although this requires more complex modeling and computation to generate results compared to empirical methods, most of this complexity is fully automated and abstracted away from the user. It therefore retains simplicity, applicability, and usability while implementing more complex modeling and more robust uncertainty estimates.

### Balance equation

The fundamental basis of the ELA model is an integrated balance formula (Eq. (1)) for steady-state glaciers from [5],

(1) $$B_{n}=\int_{0}^{L}\dot{b}dx=\beta \int_{0}^{L}\left[w\left(x\right)\left(H(x)+z(x)-ELA\right)\right]dx$$

where $B_{n}$ is the total net balance, x is the distance down glacier, $\dot{b}$ is the specific balance rate at x, L is the glacier length, β is the balance gradient, w(x) is the glacier width at x, H(x) the ice thickness at x, z(x) represents the valley topography, and ELA is the equilibrium line altitude. In steady state conditions (like we assume for glaciers with well-developed moraine sequences), the total net balance is zero. The balance gradient β can be dropped in this case, and Eq. (1) can then be adapted to solve for the ELA (Eq. (2)).

(2) $$ELA=\frac{\int_{0}^{L}w\left(x\right)H\left(x\right)dx+\int_{0}^{L}w\left(x\right)z\left(x\right)dx}{\int_{0}^{L}w\left(x\right)dx}$$

We then estimate each of the three components (H(x), w(x), and z(x)) along the length of the glacier and solve for each component using trapezoidal numerical integration to derive an estimate for ELA. Methods for the estimation of each of these components are detailed below.

### Glacier bed modeling

**Fig. 2:**
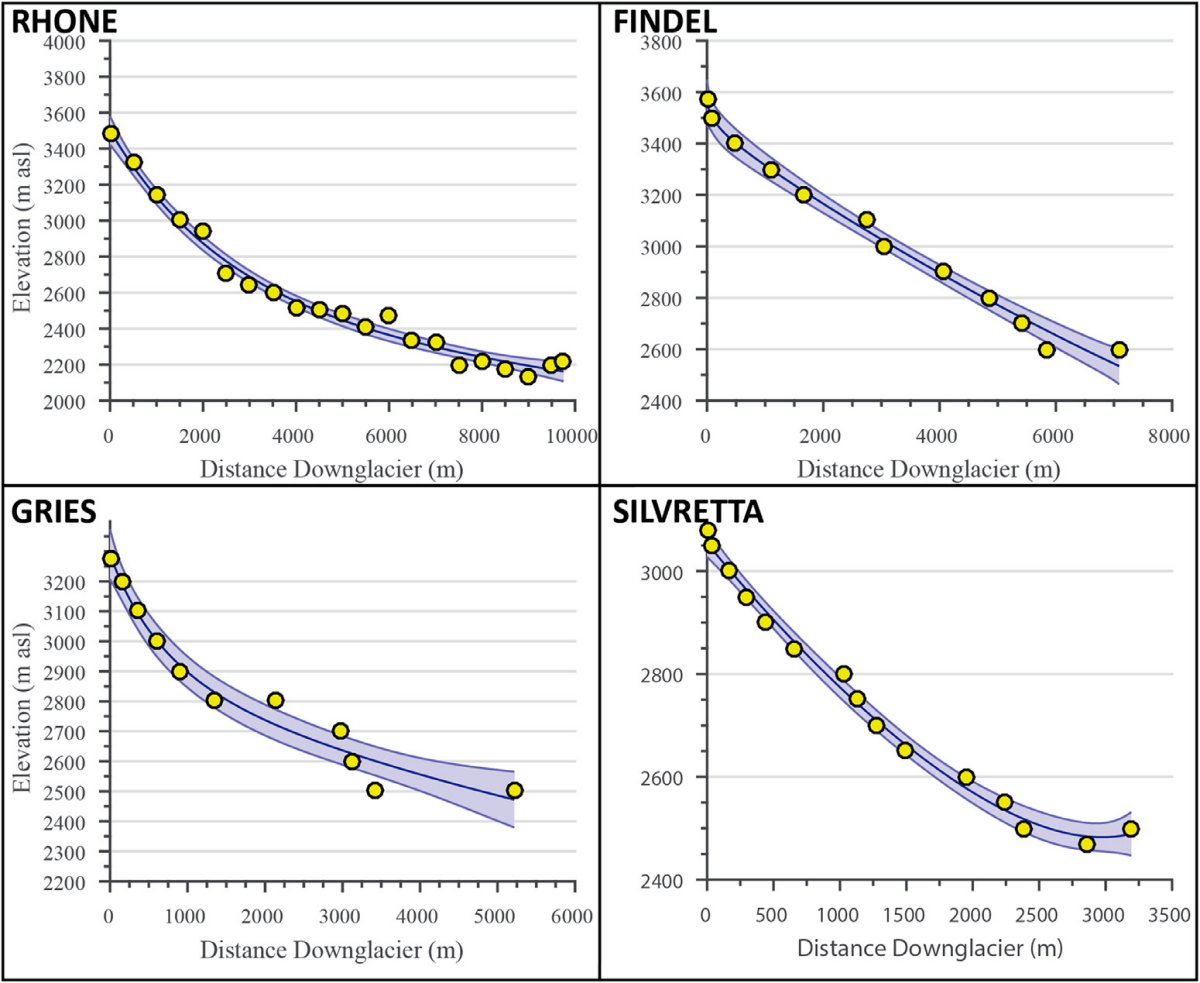
Bed elevation reconstructions for the four validation sites. Yellow circles denote measured bed elevation values, black lines represent the modeled bed profile, and blue shading represents model error (2 standard deviations). Note that scales are not consistent between subplots.

Bed topography measurements follow a representative 1D line along the glacier profile taken down the center of the glacier. We then estimate z(x) from a best-fit two-term exponential curve of this 1D profile line (Eq. (3)), where a, b, c, and d are fitting coefficients optimized in the model using the elevation data inputs (see Fig. 2 for examples). Optimizations in this ELA model use nonlinear least squares regression based on *trust region* algorithms [9].

(3) $$z\left(x\right)=ae^{bx}+ce^{dx}$$

This two-term exponential estimate is best suited for valleys with relatively simple bed topographies. Caution should be used when applying this method to glacier beds with more complex bed features, such as steep cliffs or over deepenings, as these are not always readily captured in the model.

### Ice thickness modeling

To first order, the thickness of a glacier depends largely on the slope and shear stress at the bed [1]. The simplest equation to approximate ice thickness is therefore

(4) $$H=\frac{\tau }{\rho g\sin\theta }$$

where H is the ice thickness (m), τ is the basal shear stress (Pa), ρ is the ice density ($\;{\text{kg/m}}^{3}$), g is acceleration due to gravity ($\;{\text{m/s}}^{2}$), and θ is the angle at the bed interface [1]. In areas with shallow slopes, however, Eq. (4) leads to ice thickness unrealistically approaching infinity. [5] demonstrates a square root relation between length and ice thickness (assuming perfect plasticity), which we incorporate into our estimates in order to address this issue.

(5) $$H_{m}=\frac{2}{3}\sqrt{\frac{\tau L}{\rho g\left(1+\sin\theta \right)}}$$

**Fig. 3:**
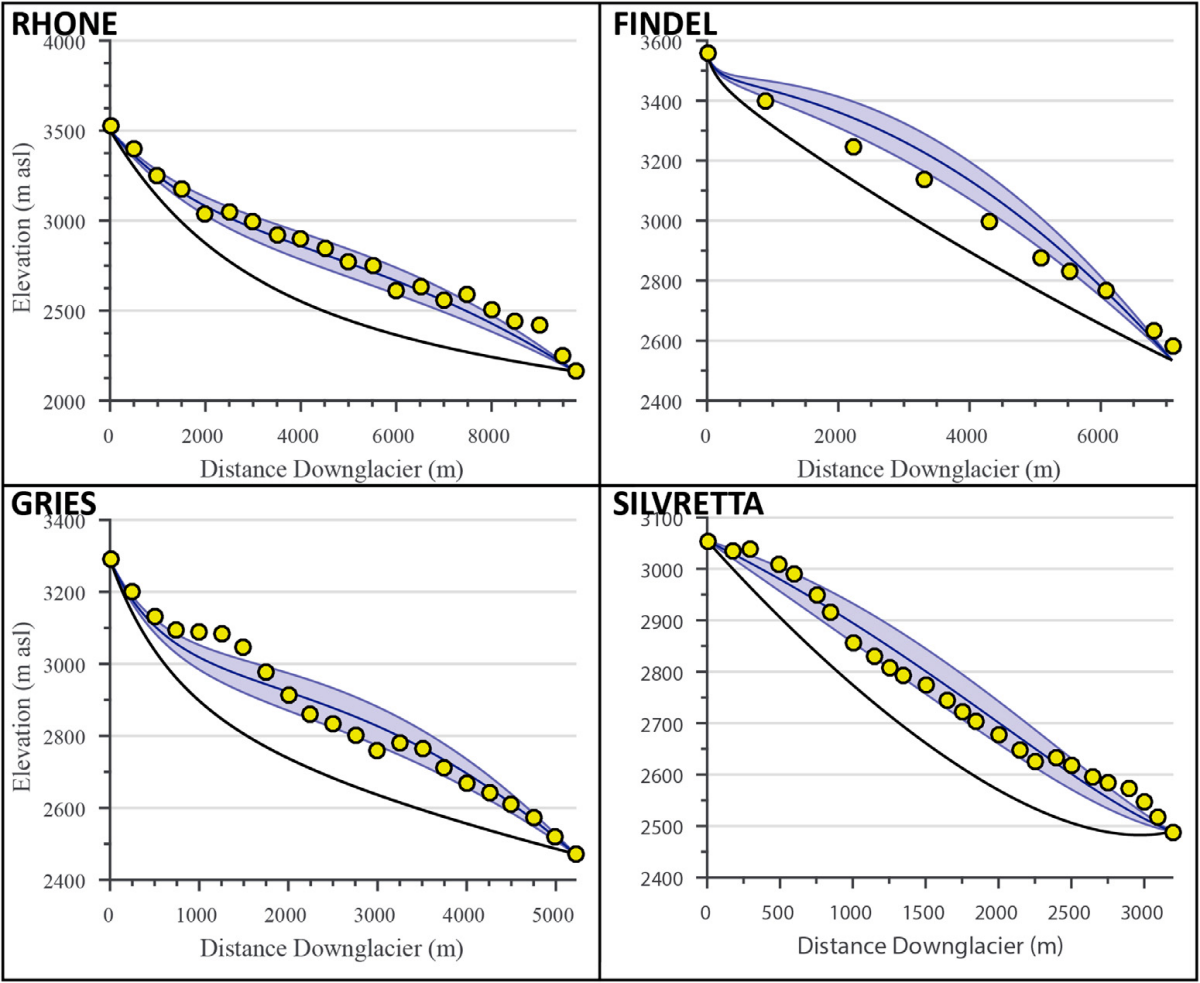
Modeled glacier ice surfaces for the four validation glaciers. Yellow circles denote measured ice elevation values, black lines represent the mean modeled bed topography (Fig. 2), blue lines represent the modeled ice surface profile, and blue shading represents model error (2 standard deviations).

Eq. (5), however, gives the mean ice thickness ($H_{m}$) for the glacier, rather than continuous values along its length. To model ice thickness profiles, we assume a parabolic distribution (true of a perfectly plastic glacier on a flat bed) around the mean ice thickness (see Fig. 3 for examples). The basal shear stress (τ) is assumed to scale with ice thickness, following the relationship presented in [10] (Eq. (6)), where Δz is the difference between the minimum and maximum bed elevation.

(6) $$\begin{array}{ccc} & & \Delta z>1600\;\text{m}⟹\tau =150\;\text{kPa}\\ & & 500\;\text{m}\leq \Delta z\leq 1600\;\text{m}⟹\tau =0.005+1.598\Delta z-0.435\Delta z^{2}\;\text{kPa}\\ & & \Delta z<500\;\text{m}⟹\tau =3\Delta z\;\text{kPa} \end{array}$$

### Glacier width modeling

Due to the high diversity in glacier shape/geometry, estimating the plan-view profile of the glacier in a consistent yet simple manner is difficult. Additionally, accurately constraining the width of the accumulation area for paleoglaciers presents further challenges, due to a lack of preserved moraines or other features delineating glacier boundaries in these areas. To best cope with these issues, we estimate glacier width using an exponential formula (Eq. (7)) of the same form as presented in [5]. The initial starting parameters are the minimum width of the glacier at the toe (w0), maximum glacier width in the accumulation zone ($w_{max}$), the distance down glacier (x), and the distance down glacier to the point of maximum width ($L_{Wmax}$).

(7) $$w\left(x\right)=w_{0}+\frac{w_{max}-w_{0}}{L_{Wmax}}xe^{1-\frac{x}{L_{Wmax}}}$$

**Fig. 4:**
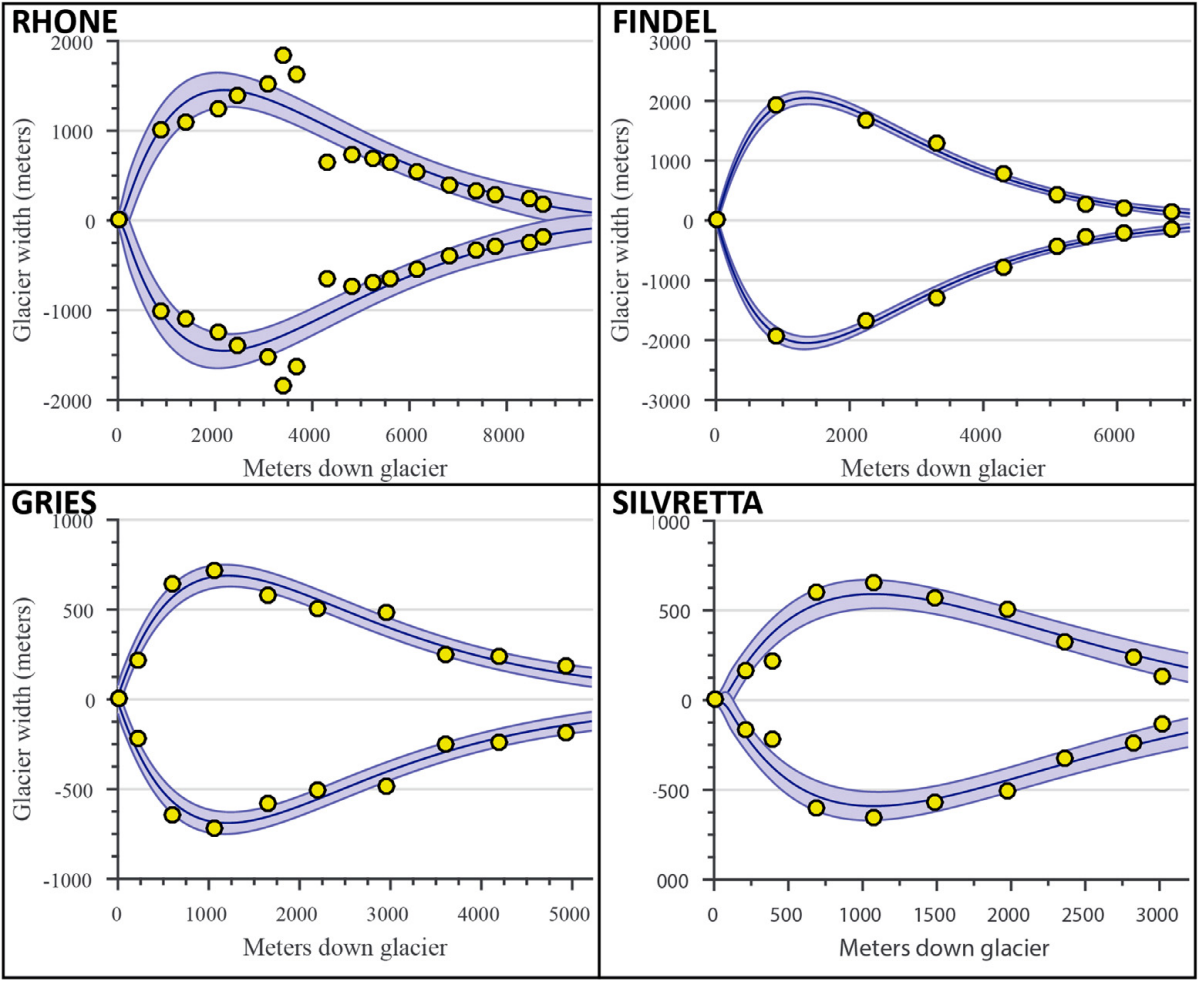
Glacier width modeling for the four validation sites. Compares the overall modeled areal profile (and modeled uncertainty) with discrete measured points of each glacier’s width. Yellow circles denote width measurements for points on the glacier, black lines represent the modeled width profile, and blue shading represents model error (2 standard deviations). Note that scales are not consistent between subplots.

This produces an exponential curve, following the general shape of many glaciers. The model then performs least squares nonlinear curve fitting (again based on trust region techniques) on the initial parameter estimates to optimize the fit to the input width estimates (see Fig. 4 for examples).

The model can also incorporate glacier tributaries. The tributaries are initially modeled as independent glaciers, including profile centerline elevations and width measurements. The calculated tributary glacier volume is then added to the main glacier at corresponding elevation levels as additional modeled glacier width. Added caution should be exercised with this model when including tributary glaciers, as the glacier plan profile can depart more severely from the assumed idealized shape constraints.

### Monte Carlo simulations

We perform Monte Carlo simulations to capture the distribution of plausible ELAs for a given glacier. Such estimation of uncertainty is important to adequately compare the significance of results, particularly if attempting to compare results from differing methodologies or between regions. Monte Carlo methods are widely used in modeling of glacier mass and energy balance for uncertainty estimation [11]. In our approach, we perform bootstrapping with replacement techniques to incorporate the uncertainty of input parameters and to include any known errors in those parameters, assuming Gaussian error distributions. Each model run consists of 1000 simulations in order to approximate a continuous distribution in plausible ELA values. The Monte Carlo simulations do increase the computational load, especially compared to the automated methods of [6], [7], taking ∼1 min to process one glacier on a single core. The model code, however, utilizes parallel processing, enabling much greater scalability to larger data sets with the proper hardware.

## Data and analysis workflow

The complete ELA model MATLAB code is publicly available (https://github.com/durbank/ELA-model), with v0.1.0 the particular version used in this manuscript. In brief, the ELA model function ELA_calc.m requires two dataset inputs (discrete estimates of bed topography and discrete estimates of glacier width, both measured downglacier along the centerline of the glacier valley) and the number of Monte Carlo simulations to perform. Approximately ten quasi-equally spaced points along the length of the glacier are often sufficient, though the optimum number depends on the length and complexity of the bed topography and glacier geometry. To avoid issues of model extrapolation (and to automatically include the overall glacier length), both the toe and the head of the glacier should be included in these measurements. The ELA model input data should be provided as a MATLAB structure with four fields, as summarized in Table 1. Tributary glaciers, if present, should be provided as variable input arguments (formatted as MATLAB structures according to Table 1) after the number of simulations to perform. The format_inputs.m function takes.csv files of glacier bed topography and glacier width measurements and creates a properly-formatted MATLAB structure to serve as input to the ELA model.

**Table 1:** Required format for ELA model inputs.

Field name	Dimensions	Field description
X_dist	[N×1]	Vector of glacier length from 0:N, where *N* is the total length of the glacier in meters
Bed_pts	[n×2]	A matrix with positions along the glacier centerline (in meters) in the first column and corresponding bed elevation measurements (meters a.s.l.) in the second
Ice_surf	[n×2]	A matrix with positions along the glacier centerline (in meters) in the first column (this should match the first column in ‘Bed_pts’) and corresponding ice surface elevation measurements (meters a.s.l.) in the second
Width_pts	[m×2]	A matrix with positions along the glacier centerline (in meters) in the first column and corresponding glacier width measurements (meters) in the second (widths should orthogonally intersect the centerline)

In addition to the inputs, there are model parameter assumptions built into the model prescribing the assumed errors for Monte Carlo sampling. Updating these assumptions to better reflect specific input data is a simple matter of editing the assigned values. Table 2 shows a summary of these parameters and their default values.

**Table 2:** ELA model error assumptions.

Variable name	Default value	Variable description
zSTD	25m	Standard deviation in measured glacier bed elevation
wSTD	60m	Standard deviation in measured glacier width values
tau_STD	$5.0\times 10^{4}\;\text{Pa}$	Standard deviation in estimated basal shear stress (used in ice thickness calculations)
rho	$917\;{\text{kg/m}}^{3}$	Density of ice (used in ice thickness calculations)
g	$9.8\;{\text{m/s}}^{2}$	Acceleration due to gravity (used in ice thickness calculations)

**Fig. 5:**
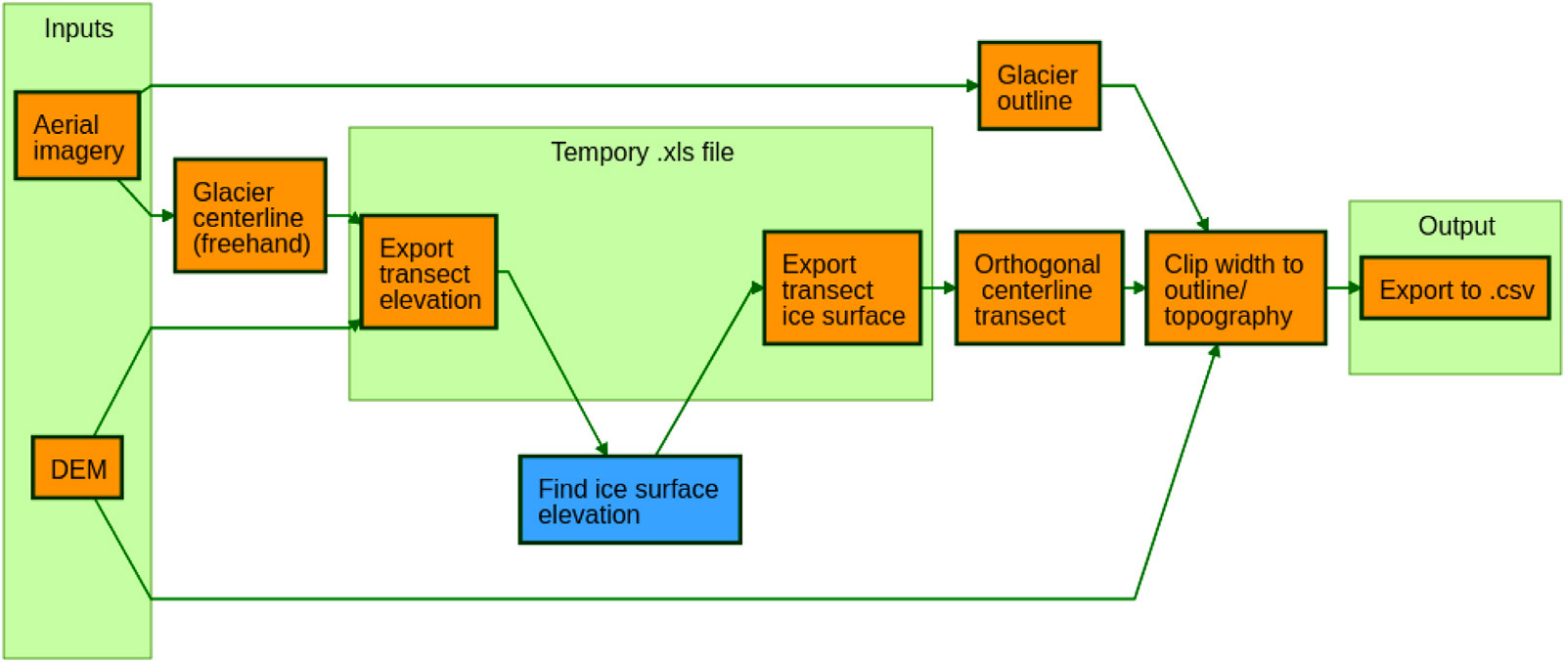
Flowchart showing the ArcGIS workflow used to generate ELA model inputs. Orange denotes steps performed in ArcMap, while blue denotes steps performed in MATLAB. The first step is to generate a characteristic centerline for the glacier. This centerline can be drawn freehand or calculated in some other way. Then extract the bed elevation along the centerline using the DEM input (recorded as distance along the centerline), and save to a temporary.xls file. Import this file into MATLAB and use the “ice_thick.m” function (part of the ELA model) to estimate the ice surface elevation at points along the centerline transect. Import the ice surface results back to ArcMap and combine with the centerline transect values. The final steps require an outline of the glacier in question. These boundaries can be drawn from the aerial imagery for modern glaciers, or else from the moraines of paleoglaciers. In the case of paleoglacier moraines, the accumulation region of the glacier can be broadly defined by the valley boundaries. Calculate polylines at each discrete point along the transect at the elevation of the ice surface and orthogonal to the centerline at that point. The intersection of these orthogonal lines (at the elevation of the ice surface at that point) with either the glacier boundaries (in the case of modern glacier outlines and portions of paleoglaciers constrained by moraines) or the bed topography (in the case of the accumulation zone of paleoglaciers) defines the glacier width at each transect point. The results of distance down the glacier centerline, estimated bed elevation, and estimated glacier widths are then saved as a.csv file for import to the ELA model.

For the development and validation of this model, we used a particular ArcGIS software workflow to generate the ELA model inputs. We include this workflow as a diagram (Fig. 5), but model inputs can be generated and provided using any desired methods, as long as they are properly formatted.

## Model validation

We validate the ELA model by matching our reconstructions with direct observations of four modern glaciers in the European Alps. These glaciers were primarily selected due to the availability of data requisite for a data-model comparison (including present-day ice thickness, bed topography beneath the present-day glacier, mass balance measurements, aerial photography and DEMs). Moreover, the validation glaciers show variation in overall area, length, width, and elevation extent, thereby providing a range of possible glacier geometries (see Table 3 for differences). This range of glacier characteristics enables a more rigorous test of robustness and general applicability of the ELA model. Although all 4 validation glaciers are limited to the European Alps, the method should be more widely applicable to similar glaciers in other regions (clean-ice, land-terminating temperate mountain glaciers) without regional tuning of empirical coefficients. The four test glaciers are the Gries, Findel, Rhone, and Silvretta Glaciers. Three of these glaciers (Gries, Silvretta, and Findel) have continuous multi-year mass balance measurements from stake networks compiled by the World Glacier Monitoring Service (WGMS), and therefore make for the most compelling comparisons. The Rhone Glacier has mass balance measurements from a handful of isolated years, providing a less certain, but still useful comparison to the model and other glaciers.

**Table 3:** Summary characteristics of the 4 validation glaciers.

Glacier	Min elevation	Max elevation	Observed ELA	Area	Length	Max width
Gries	2500m	3277m	3084m	$4.28\;{\text{km}}^{2}$	5216m	1441m
Silvretta	2498m	3078m	2829m	$2.58\;{\text{km}}^{2}$	3193m	1307m
Findel	2600m	3570m	3233m	$14.5\;{\text{km}}^{2}$	7089m	3835m
Rhone	2200m	3480m	2918m	$14.4\;{\text{km}}^{2}$	9751m	3256m

### Data sources

We obtained width and overall length measurements for the 4 validation glaciers from LANDSAT 5 satellite imagery and ASTER global digital elevation models (GDEMs). As the LANDSAT 5 imagery has a horizontal resolution of ±30 m, we prescribe a conservative ±60 m error for glacier width measurements (error for both edges of glacier boundaries). ASTER GDEM data have a vertical root-mean-squared error of ±15 to ±25 m, depending on several environmental conditions (surface covering, topography, surface roughness, etc.). [12]. As our model exclusively involves mountainous snow-covered regions, we utilize the more conservative ±25 m error in calculations of bed topography and ice surface elevations. Bed elevation validation measurements are from modeled topographies in [13], [14], constrained using multiple GPR profiles and/or borehole depths for each glacier.

The Silvretta and Gries glaciers have the best-constrained mass balances with  50 years of published data for each [15]. In order to compare the current climatic ELA of these glaciers with our modeled ELA, we determine measured ELAs from the linearly detrended, annually-measured ELA values from 1981 to 2010 for both glaciers, with uncertainty calculated using a 95% margin of error. The Findel Glacier has similarly well-constrained mass balance measurements from a glacier stake network [15], but with a much shorter record (2005–2010). We compare the mean ELA over this time to the modeled ELA for Findel Glacier. The Rhone Glacier does not have consistent year-to-year mass balance measurements. Instead, we take modeled steady-state ELA estimates from air temperature correlations (1971–1990) provided in [4]. These ELA estimates are constrained with the few years of available stake mass balances (mean $r^{2}$ between measured ELA and air temperature-correlated ELA is 0.89). No uncertainty estimates were provided for the Rhone Glacier ELA. For consistency, we assume Gaussian uncertainties with bounds similar to the margins of error of the mass balances for the Silvretta, Gries, and Findel glaciers (±50 m).

### Model comparisons

**Fig. 6:**
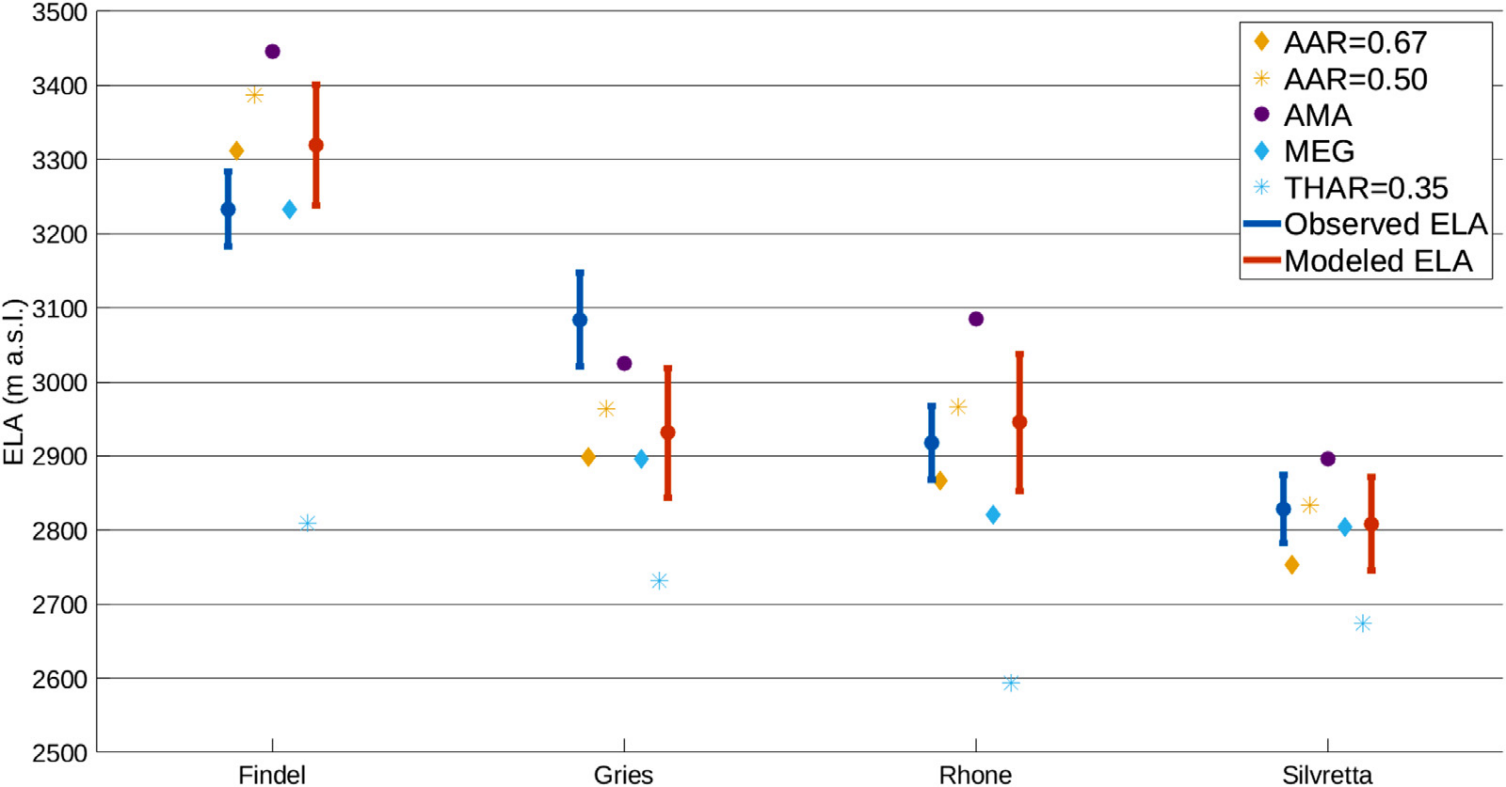
Comparison of observed ELA values, ELA estimates using the outlined ELA model, and estimates from several empirical methods (AAR, AMA, MEG, and THAR) for the four validation glaciers. Modeled and measured estimates are within the margins of error for the 4 validation glaciers, although Gries Glacier appears to have a systemic bias. The results show a mean bias in central estimates for modeled ELAs of −14.8 m relative to measured ELAs, with no consistent direction in bias. The largest difference occurs with the Gries Glacier, with a central bias in modeled ELA of −155 m relative to the observed ELA.

The model results, including bed topography, ice thickness, plan-profiles, and ELAs, are summarized in Fig. 2, Fig. 3, Fig. 4 and 6 for all four validation sites. Most of the intermediate model outputs match measured values within error. Points of increased disagreement likely result mainly from local variability and the inherent smoothing caused by model fit constraints and optimization. Exceptions to this include the overdeepened section apparent in the Gries Glacier (Fig. 2), which represents a systemic shift in bed topography not adequately captured in the model. Similarly, most differences in modeled and measured ice surface (Fig. 3) likely result from local variations in ice thickness of a scale finer than the input data resolution (e.g. ice crevasses), but with little effect on the final ELA estimate. An exception to this explanation is Findel Glacier, wherein the model appears to systemically overestimate the ice thickness, and a corresponding overestimation of the ELA by a similar magnitude (see Figs. 3 and 6). Although isolating an exact reason for this overestimation is challenging, it may be related to violations of the assumed perfect plasticity of the modeled ice or to ice thinning/downwasting due to climate disequilibrium, neither of which are accounted for in this ELA model. Modeled glacier width results generally closely match those recorded from satellite imagery (Fig. 4). The most noticeable exception to this is the Rhone Glacier, with a few clear outliers in the accumulation area. These may be related to difficulties in accurately defining the glacier boundaries in the accumulation area, or else may simply represent a more complex glacier geometry that this ELA model will not fully capture. Regardless, these deviations do not appear to significantly affect the final ELA estimate.

Modeled ELA estimates for the four validation sites and comparisons to corresponding observed ELAs are presented in Fig. 6. The figure additionally includes empirical estimates of ELA using the AAR, AMA, MEG, and THAR methods for each validation glacier. We use two AAR values (0.5 and 0.67) to show how the choice of this parameter influences the estimate. We assign THAR a coefficient of 0.35. As the MEG is equivalent to THAR=0.5, these two metrics also show the effect of varying this ratio as well. All four validation glaciers show agreement within errors between observed and modeled ELA values, although the central estimate for Gries glacier differs by ∼150 m. None of the empirical methods consistently estimate the ELA within observed uncertainty for all 4 glaciers, although the AAR=0.50 and MEG iterations perform the best of the empirical methods. THAR (with ratio=0.35) consistently underestimates the ELA, while AMA consistently overestimates the ELA for these glaciers. Table 4 summarizes the the mean biases over the four validation sites compared to the observed mean ELA. The ELA model and the AAR method (ratio=0.50) perform the best overall with mean biases of −14.8 m and 21.8 m respectively.

**Table 4:** ELA mean biases for different methods.

Method	Mean bias (m)
AAR=0.50	21.8
AAR=0.67	−58.3
AMA	96.8
MEG	−77.1
THAR=0.35	−314
ELA model	−14.8

Likely sources of error to explain discrepancies between the observations and modeled results involve more complex considerations not accounted for with the simple ELA model. For instance, more complex bed topographies, differences in shading/shielding by valley walls, debris cover, and accumulation through avalanching can all affect the recorded ELA in mass balance measurements, none of which are considered in the ELA model. It is important to note that the model is particularly sensitive to errors in bed topography, as these values influence estimates of slope, ice thickness, and width and therefore can potentially strongly affect the final ELA estimates. Differences in steady-state assumptions may also be an important factor in differences between modeled and measured modern ELAs. The ELA model assumes steady-state conditions, whereas the annual mass balance reflects emergent climate conditions. Glaciers typically have either an annual mass surplus or deficit in a given year, complicating comparisons of our results to measured ELA values. Such a limitation, however, is inherent to all morphology-based ELA models. For instance, all methods significantly underestimate the ELA for Gries glacier, suggesting this glacier could be strongly out of equilibrium. Overall, the presented results show a high degree of confidence in the model’s ability to estimate glacier ELAs (within calculated uncertainties) from relatively few geomorphic inputs, supporting the use of the presented ELA model for simple valley glaciers across a wide spectrum of bed slope geometries, glacier shapes, glacier widths, and elevation extents.

The incorporation of additional variables and modeling components could address some of these limitations and improve the overall effectiveness of the model. For instance, the methods presented in [6], [8] also include a “shape friction factor” parameter that accounts for lateral drag in topographically-constrained glacier valleys. This *F* factor relates the frictional lateral drag to the glacier cross-sectional area and perimeter length of the ice-topography contact [8]. As these required inputs are also generated during intermediary steps in our ELA model, an *F* factor implementation could be incorporated into the ELA model in the future, potentially improving the ELA estimates for valley glaciers. In light of the sensitivity of the model to bed slope, using a non-parametric interpolation method for bed topography estimates could improve the ELA estimates, but would also require more complex modifications of the model to avoid discontinuous step changes in ice thickness and other parameters.

## Conclusions

The model described here accurately estimates ELAs from Alpine valley glaciers of varying size, topography, and areal distribution while utilizing a small set of easily-obtained measurements. The model provides errors based on the physical uncertainties of model inputs, a crucial factor for determining the significance and importance of results. We validate the model on a set of glaciers in the Alps spanning a variety of characteristics (bed topography, size, elevation extent, etc.). The model performs at least as well as traditional empirical methods of ELA estimation while minimizing reliance on optimized empirical coefficients, adding uncertainty estimates, and providing insight for the sensitivity of individual glaciers to model inputs. Based on these validations and the physics-based nature of the model, this ELA model should serve as a robust, easily applicable, self-consistent method for ELA glacier reconstructions in varied areas, including the broader European Alps, alpine regions of the Arctic, the Southern Alps in New Zealand, and similar glaciated locations. The model should also be readily applicable to paleoglacier reconstructions based on preserved moraine sequences, permitting rapid and consistent comparisons of glacier changes through time and across diverse regions. Such studies will permit enhanced insight into the mechanisms of climate change in the past, and help us to better understand present and future changes to critical glacial and water resources in a warming world.

## Declaration of Competing Interest

The Authors confirm that there are no conflicts of interest.
